# Intranasal Oxytocin for Alcohol Use Disorder: A Randomized, Double‐Blind, Placebo‐Controlled Multisite Trial Assessing Efficacy and Safety

**DOI:** 10.1111/acer.70326

**Published:** 2026-07-15

**Authors:** Nassima Ait‐Daoud Tiouririne, Jospeh A. Legan, Eric Devine, Kristen Lee, Elise M. Weerts, Jennifer D. Ellis, Lara Ray, Steve Shoptaw, Daniel Falk, Megan Ryan, Raye Litten, Chamindi Seneviratne, Janet Ransom, Charles Scott

**Affiliations:** ^1^ Center for Leading Edge Addiction Research University of Virginia Charlottesville Virginia USA; ^2^ Department of Psychiatry Boston University Chobanian & Avedisian School of Medicine Boston Massachusetts USA; ^3^ Section of General Internal Medicine, Department of Medicine Boston University School of Medicine and Boston Medical Center Boston Massachusetts USA; ^4^ Department of Psychiatry and Behavioral Sciences Johns Hopkins University School of Medicine Baltimore Maryland USA; ^5^ Department of Psychology University of California Los Angeles Los Angeles California USA; ^6^ Department of Psychiatry and Biobehavioral Sciences University of California Los Angeles Los Angeles California USA; ^7^ Department of Family Medicine and of Psychiatry and Biobehavioral Sciences David Geffen School of Medicine at UCLA Los Angeles California USA; ^8^ National Institute on Alcohol Abuse and Alcoholism Bethesda Maryland USA; ^9^ Fast Track Drugs and Biologics North Potomac Maryland USA

**Keywords:** alcohol use disorder, clinical trial, medication development, oxytocin

## Abstract

**Background:**

The neuropeptide oxytocin offers a potentially effective treatment for alcohol use disorder (AUD). Acting centrally in the brain, oxytocin mediates a range of behavioral effects, including reward, stress response, social affiliation, learning, memory, and addictive behaviors. Animal studies demonstrate oxytocin's ability to reduce alcohol consumption, but results in human trials have been mixed. Some studies report reductions in withdrawal symptoms, heavy drinking, and craving, whereas others show no effects. The purpose of this 12‐week clinical trial was to evaluate the efficacy and safety of intranasal oxytocin in individuals with AUD.

**Methods:**

Oxytocin (up to 70 International Units [IU]/day) or placebo was administered to 100 individuals diagnosed with AUD in a double‐blind, randomized, 12‐week multisite trial. The primary outcome was the weekly percentage of heavy drinking days (PHDD) over 10 weeks of maintenance treatment. Secondary outcomes included additional drinking measures, urge to drink, psychological assessments, alcohol‐related consequences, nicotine use, and safety and tolerability of intranasal oxytocin.

**Results:**

No significant differences were observed in PHDD between groups during the 10‐week maintenance phase. However, a small, numerically greater reduction in drinking was seen in the oxytocin group compared with placebo from weeks 9 to 12. Similar patterns were found across secondary drinking outcomes. No significant differences were observed in AUD symptoms, alcohol‐related consequences, craving, mood, sleep, pain, or substance use. At the end of treatment, participants receiving oxytocin scored significantly lower on measures of anger and physical aggression. Oxytocin was well tolerated; adverse events were mild and comparable across groups. The most common event in both groups was hyposmia.

**Conclusion:**

Intranasal oxytocin was well tolerated but did not significantly reduce drinking or AUD‐related outcomes. Higher doses, longer treatment duration, or larger trials may be required to identify responsive subgroups and determine oxytocin's efficacy in AUD.

**Trial Registration:**

ClinicalTrials.gov, Identifier: NCT03878316. Registered March 14, 2019 (https://clinicaltrials.gov/ct2/show/NCT03878316)

## Graphical abstract

Intranasal oxytocin was safe but did not significantly reduce drinking in a 12‐week randomized trial of alcohol use disorder. A late trend toward reduced drinking highlights the need for optimized dosing, longer treatment duration, and identification of responsive subgroups.

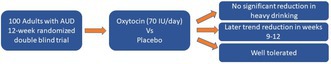

## 1 Introduction

Alcohol use disorder (AUD) affects over 28.9 million Americans and is a leading contributor to medical, psychological, and economic burdens (Substance Abuse and Mental Health Services Administration 2024). Currently, the U.S. Food and Drug Administration (FDA) has approved three medications for the treatment of AUD—naltrexone, acamprosate, and disulfiram (Witkiewitz et al. 2019). Several repurposed medications also have shown efficacy in reducing drinking in AUD‐diagnosed individuals (Litten et al. 2018). However, because of AUD's significant heterogeneity, these medications do not work for everyone. Continued investigation of new medications is vital to give clinicians more choices for helping people suffering from this devastating disorder.

Oxytocin is a promising compound for treating AUD. This 9‐amino‐acid polypeptide hormone is synthesized in the paraventricular and supraoptic nuclei of the hypothalamus and released by the posterior pituitary into general circulation (Lee et al. 2016). It acts peripherally to promote uterine contraction and lactation. Preclinical studies have shown that oxytocin also acts centrally on numerous cortical, limbic, and basal ganglia structures to mediate an array of behavioral effects, including reward, stress response, social affiliation, learning, and memory (Lee and Weerts 2016; Lee et al. 2016). Oxytocin's potential for treating addictive disorders was confirmed in preclinical studies, where it reduced alcohol intake in a variety of animal models (Ryabinin and Fulenwider 2021). In addition to reducing alcohol consumption, oxytocin was found to reduce motivation to consume alcohol in rat models (Tunstall et al. 2019). Oxytocin also reduced stress‐induced alcohol relapse in a mouse model of AUD and posttraumatic stress disorder (PTSD), suggesting a role for it in treating people with this comorbidity (Becker et al. 2023). However, oxytocin's effectiveness for treating AUD in human studies has been mixed (Ryabinin and Fulenwider 2021). Pedersen et al. (2013) and Pedersen (2017) found that oxytocin was effective in reducing alcohol withdrawal and heavy drinking in AUD individuals. Mitchell et al. (2016) found that oxytocin improved social perception and reduced cue‐induced alcohol craving in participants diagnosed with alcohol abuse and anxiety. In contrast, Flanagan et al. (2019) and Stauffer et al. (2022) found no effect of oxytocin on alcohol craving among individuals with AUD and PTSD, while Melby et al. (2021) found no effect on alcohol consumption. Additional studies provide further insights into oxytocin's role in AUD. Bach et al. (2019) found that oxytocin significantly reduced nucleus accumbens (NAc) functional connectivity with brain regions associated with visual processing (e.g., cuneus) and attention to alcohol‐related cues. Lee et al. (2016) reported that oxytocin may modulate stress‐induced alcohol seeking and social behavior in individuals with AUD, highlighting its potential in addressing the social dysfunction component of addiction. Many of these oxytocin human studies had limitations, including small or non‐treatment‐seeking samples and variations in oxytocin dosing, which may contribute to inconsistent findings.

In the present study, a larger population of treatment‐seeking AUD participants was recruited using a higher dose of oxytocin compared to most of the above studies. The primary objective of this study was to evaluate the efficacy of intranasal oxytocin in reducing alcohol use in heavy drinkers with AUD during 12 weeks of treatment. The primary a priori outcome was weekly percentage of heavy drinking days (PHDD) over the 10 weeks of maintenance treatment. The study also examined oxytocin's effects, compared with placebo, on the number of AUD symptoms, alcohol negative consequences, urge to drink, mood endpoints, sleep, pain, THC use, smoking, nicotine use, and safety and tolerability.

## 2 Methods

### 2.1 Study Population

Randomized participants (*n* = 100) were diagnosed with (at least) moderate AUD (i.e., meeting 4 or more criteria in the past year), according to the *Diagnostic and Statistical Manual*, 5th edition (DSM–5) (American Psychiatric Association 2013). Eligible participants were 21 years of age or older; reported drinking an average of at least 21 standard drinks per week for women or 28 standard drinks per week for men; and had at least one heavy drinking day per week during the 28‐day period before giving consent. Participants had not been diagnosed with a current substance use disorder (other than alcohol or nicotine) or major psychiatric disorder (e.g., psychotic, bipolar, and eating disorders; major depressive episode). See Appendices S1 and S2 for full inclusion/exclusion criteria and assessment schedule, respectively.

### 2.2 Study Design

The study was a randomized, double‐blind, placebo‐controlled, 12‐week outpatient treatment clinical trial. Candidates were treatment‐seeking volunteers who responded by telephone to advertisements from four US academic sites. Participants were compensated for their time and travel.

Eligibility was established during the initial screening and baseline visit. During weeks 1–12 of the treatment phase, participants were seen during eight in‐clinic visits and five telephone visits. There was a final clinic visit at the beginning of week 13 and a follow‐up telephone interview at week 14 (approximately 1–2 weeks after the last in‐clinic study visit) to assess medication safety and changes in drinking. A breath alcohol concentration ≤ 0.02% was required to complete the in‐clinic assessments.

Participants were randomly assigned in a 1:1 ratio to receive either intranasal oxytocin or matched placebo, using a permuted block technique stratified by location. Clinical site was used as the stratification variable to compensate for differences in the local study populations and investigative staff that might influence a participant's drinking behaviors and impact endpoints. Randomization was implemented via a centralized, interactive web‐based response system (IWRS).

The study adhered to the Declaration of Helsinki and Good Clinical Practice guidelines of the International Conference on Harmonization. All patients gave voluntary, written informed consent prior to the initiation of any study procedures. The protocol, consent, and all study‐related materials were reviewed and approved by a central institutional review board and the FDA. An independent data and safety monitoring board reviewed patient safety data throughout the study.

### 2.3 Investigational Products

Investigational products were manufactured by the University of Iowa and packaged and distributed in drug kits by Catalent Inc. Both oxytocin (70 IU/mL concentration) and the placebo were packaged in 20 mL vials with a P270 pump spray actuator. The oxytocin pump spray actuator delivered 100 μL in a single spray (equivalent to 7 IU).

Participants received either oxytocin or placebo once daily (morning) for 2 weeks (Weeks 1 and 2) and then twice daily (morning and evening) for 10 weeks (Weeks 3–12). For each treatment, participants alternated nostrils, insufflating a total of five doses (100 μL oxytocin) at 30‐s intervals. The total dose was 500 μL or 35 IU of oxytocin each day for 2 weeks, then twice a day for 10 weeks, with a total daily dose of 70 IU of oxytocin. Participants who could not tolerate the target dose were permitted dose reductions by 14 IU or 200 μL increments per dose (2 less sprays).

Intranasal oxytocin has been studied in small clinical trials of participants with mental health disorders at doses ranging from 18 to 320 IU given as single doses or in divided doses given BID or QID for as long as 13 weeks. The dose of 70 IU/day for the current study was selected based on two small randomized controlled trials (Feifel et al. 2010; Ohlsson et al. 2005) showing that doses of intranasal oxytocin up to 80 IU/day were safe and effective at reducing alcohol withdrawal symptoms, very heavy drinking, and anxiety in individuals with AUD.

### 2.4 Behavioral Platform

Participants viewed *Take Control*—a computerized bibliotherapy platform (Devine et al. 2016) derived from the National Institute on Alcohol Abuse and Alcoholism's (NIAAA's) self‐help approach, *Rethinking Drinking* [2009]. *Take Control* consists of seven modules with a run‐time of approximately 10 min per module. Participants were asked to view a single module at each clinic visit. Any missed modules, due to a missed clinic visit, were viewed at the next visit.

### 2.5 Measures of Efficacy and Assessments

Alcohol consumption was captured via the timeline follow‐back and Form 90 interview methodology and procedures (Miller 1996; Sobell and Sobell 1992). Drinks were converted into standard drink units (1 standard drink = 0.6 oz. of pure alcohol) for all subsequent analyses. The a priori primary efficacy endpoint was the percent of heavy drinking days (PHDD) (Falk et al. 2010) during the 10‐week maintenance treatment period (Study Weeks 3–12). A “heavy drinking day” was defined as four or more drinks per drinking day for women or five or more drinks per drinking day for men.

A priori secondary efficacy endpoints featured other drinking measures, including percent of days abstinent, drinks per week, drinks per drinking day, percent of participants who were abstinent, percent of participants with no heavy drinking, and percent of participants with reduced drinking risk (i.e., at least a 1‐ or 2‐level reduction in World Health Organization risk drinking categories) (Witkiewitz et al. 2017).

Secondary outcomes in the trial included the following. Urge to drink alcohol was assessed at baseline and at scheduled visits until the end of study using the Urge‐to‐Drink Questionnaire (USD) (Flannery et al. 1999). The Profile of Mood State (POMS), capturing dimensions of affect or mood (McNair and Heuchert 2005), and the Patient‐Reported Outcomes Measurement Information System (PROMIS), measuring alcohol‐related consequences, sleep disturbances, and pain interference (Pilkonis et al. 2016), were recorded at baseline and during the study. DSM–5 AUD was assessed at the beginning and end of treatment using the Mini Neuropsychiatric Interview (MINI) (Sheehan et al. 1998). The Experiences in Close Relationships‐Relationship Structure Questionnaire (ECR‐RS), assessing two‐dimensional relationship‐specific attachment structure, was administered during the study (Fraley et al. 2011). An Inventory of Drinking Situations (IDS‐30), measuring reward and relief drinking, was conducted at baseline (Mann et al. 2018). A smoking nicotine‐use interview was performed at baseline. The interview included three questions to assess nicotine use (i.e., cigarette smoking or other products) during the study: (1) Over the past week, on how many days did you smoke cigarettes?; (2) on the days you smoked during the past week, how many cigarettes did you smoke on average?; and (3) over the past week, on how many days did you use other nicotine products (e.g., chew, cigars, cigarillos, e‐cigarettes, vape, gum, patch). THC use was measured via a positive urine weekly for the first 4 weeks and then biweekly until the end of study. The Spielberger State–Trait Anxiety Inventory (STAI) (Spielberger et al. 1983) and Barrett Impulsiveness Scale (BIS‐11) (Patton et al. 1995) were measured at baseline. Drinking goal was assessed at baseline and ascertained whether participants wanted to be totally abstinent or reduce drinking at the end of the study.

### 2.6 Compliance and Retention

Medication compliance (defined as the average amount of investigational product taken versus the total amount prescribed, overall and weekly) was based on the participant's self‐report of number of sprays taken, in both the oxytocin and placebo groups. In addition to self‐report, each bottle of investigational product was weighed prior to dispensing to the participant and again after use to determine the amount removed. The estimated percentage of prescribed dose received was calculated based upon the product taken divided by the number of days the product was prescribed (1 g of drug was prescribed per day during the maintenance period). The research participation rate, defined as the percentage of participants with complete drinking data, was compared between treatment groups. The percentage of participants discontinuing medication or withdrawing early from the study also was recorded.

### 2.7 Safety Assessments and Adverse Events (AEs)

Safety assessments were taken both at baseline and during treatment. Safety was assessed via vital signs; blood chemistry tests; urine tests for illicit drug use; blood alcohol concentration, as measured by breathalyzer; AEs; concomitant medication use; cardiac conduction, measured by electrocardiogram; alcohol withdrawal, measured by the Clinical Institute Withdrawal Assessment for Alcohol–revised (CIWA‐Ar) (Sullivan et al. 1989); and suicidal ideation, measured by the Columbia Suicide Severity Rating Scale (Posner et al. 2011). Other mood measurements included the Buss Perry Aggression Questionnaire (BPAQ‐SF), which was given during the study to assess physical aggression, verbal aggression, anger, and hostility (Buss and Perry 1992; Diamond and Magaletta 2006).

Because the study formulation of oxytocin was administered intranasally, the University of Pennsylvania Smell Identification Test (UPSIT) was used in entry criteria to include only individuals with non‐impaired sense of smell and was repeatedly administered during the study to capture changes in olfactory ability (Ditraglia et al. 1991). Nasal erosion was also assessed through direct examination at baseline and during the study.

The Simplified Nutritional Appetite Questionnaire (SNAQ), which measures appetite and predicts weight loss, was administered during the study because oxytocin is known to reduce appetite, body weight, and reward‐ and hunger‐driven eating. An SNAQ score ≤ 14 indicates a significant risk (at least 5%) for weight loss within 6 months (Wilson et al. 2005).

Subjective AEs were assessed in the clinic and during telephone interviews. These included the open‐ended question: “How have you been feeling since your last visit?” Participants also reported concomitant medication use. AEs were coded using preferred terms from the Medical Dictionary of Regulatory Activities (MedDRA) and grouped by system, organ, and class (SOC) designation. Each recorded AE was counted one time for a given participant. If the same event occurred on multiple occasions, the highest severity was assumed.

### 2.8 Statistical Analysis

The full analysis set included all participants who were randomized to take part in the study and who took at least one dose of investigational product. This full analysis set was used to evaluate all efficacy and safety endpoints.

All efficacy outcomes were assessed during the maintenance treatment period (weeks 3–12). The primary a priori outcome was weekly percentage of heavy drinking days (PHDD) over the 10 weeks of maintenance treatment.

Continuous outcomes were measured at multiple time points and analyzed using a repeated‐measures mixed‐effects model. Least square means, standard errors (SEs), and 95% confidence intervals (CIs) are presented for each treatment group and were derived from fully adjusted models on untransformed outcomes (to facilitate clinical interpretation), averaged across the maintenance period. Cohen's *d* and *p*‐values were based on the fully adjusted models with the appropriately transformed outcome variables (if skewed).

For dichotomous outcomes, unadjusted prevalence rates were determined during the maintenance period. Adjusted odds ratios (aORs) and *p*‐values were derived from fully adjusted logistic regression models; the number of covariates was limited by the number of events for each dichotomous outcome (Peduzzi et al. 1996).

Exploratory moderation analyses were conducted for PHDD outcome during the maintenance period to evaluate whether a differential treatment effect existed for 13 patient characteristics of theoretical and scientific interest (see Appendix S3 for a listing of the moderators and a description of the analytic procedure). These characteristics were measured at baseline and included: drinks per week, number of AUD symptoms (MINI), IDS (relief and reward drinking subscales), gender, attachment‐related anxiety (ECR‐RS), POMS subscales of depression–dejection and tension–anxiety, STAI, BIS (self‐control and impulsive behavior subscales), history of alcohol withdrawal (MINI), and drinking goal.

Except for the primary outcome, no imputation was performed for missing data in the tabled model results. However, as a sensitivity analysis, models were re‐estimated with imputation for missing data. For all statistical tests, *p* < 0.05 (2‐tailed) was considered statistically significant. No adjustment was made for multiple inferential tests. For the primary outcome (PHDD), 80% power was estimated to detect a treatment effect (Cohen's *d*) of 0.60, given a two‐sided significance level of 0.05, and an initial 100 participants randomized with projected 12% attrition, yielding 88 participants completing the trial (44 per arm). Data were analyzed with SAS version 9.4 (SAS Institute, Cary, NC). See Appendix S3 for details regarding the statistical analyses.

### 2.9 Stopping Guidelines

Enrollment would be suspended if more than two treatment‐related serious adverse events (SAEs) occurred. In such an event, an independent Data and Safety Monitoring Board (DSMB) would review safety data and, in consultation with the sponsor (NIAAA), determine whether the trial should be terminated. The NIAAA or FDA could also stop the study at any time for safety concerns. No interim analyses for efficacy or futility were planned.

#### 2.9.1 Protocol and Statistical Analysis Plan

The full study protocol and statistical analysis plan are available on ClinicalTrials.gov (NCT03878316).

## 3 Results

### 3.1 Participant Characteristics

**FIGURE 1:**
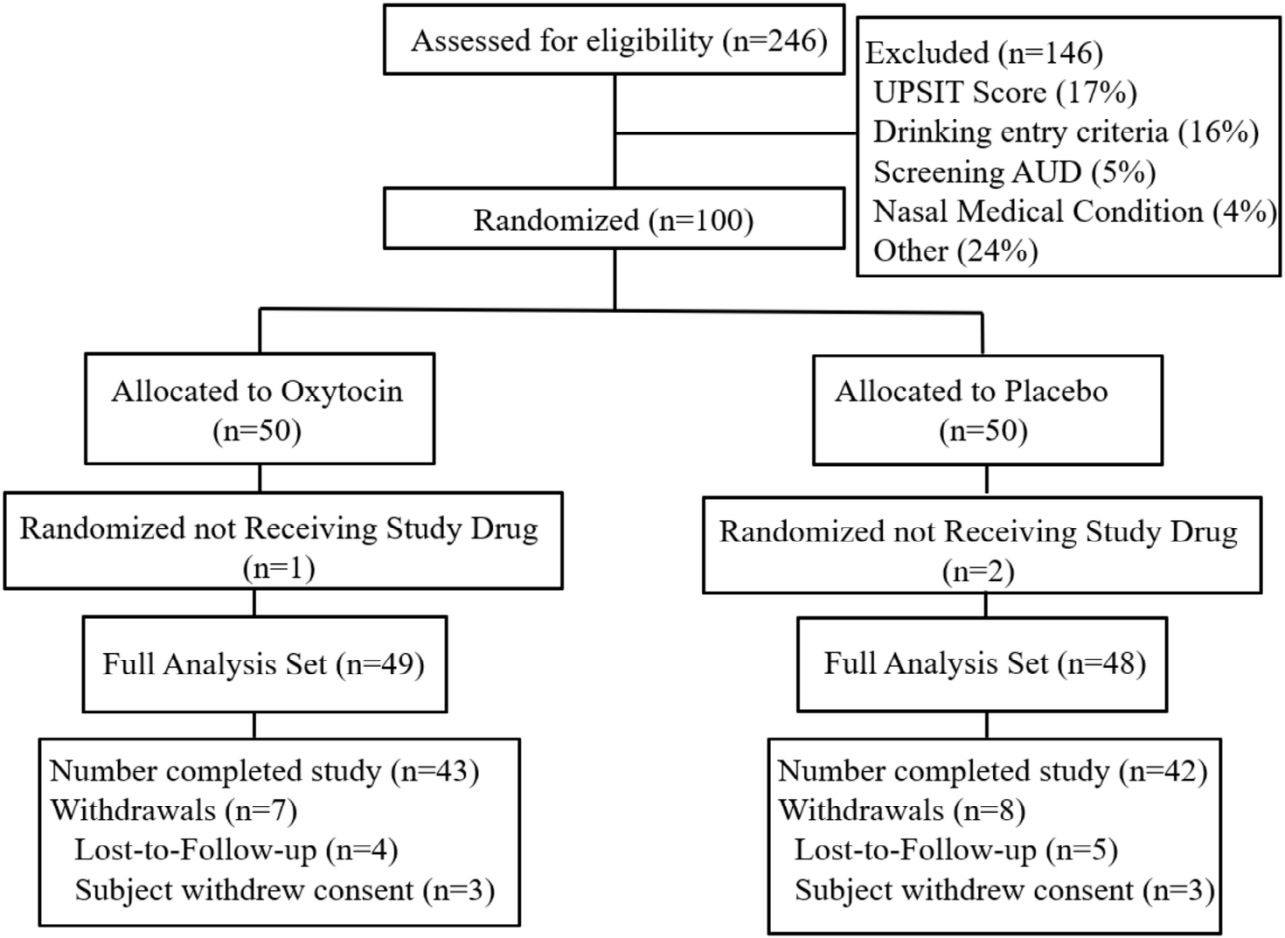
CONSORT flow diagram showing participant enrollment, randomization, allocation, and study completion. Of 246 assessed for eligibility, 100 were randomized to receive oxytocin (*n* = 50) or placebo (*n* = 50). Numbers of participants who did not receive the study drug, completed the study, and withdrew (with reasons) are shown for each group.

The overall disposition of study participants is shown in Figure 1. A total of 246 individuals consented to the study. Of these, 100 participants were eligible and randomly assigned to receive either oxytocin (*n* = 50) or placebo (*n* = 50). Three participants (*n* = 2 placebo and *n* = 1 oxytocin) did not return after randomization, resulting in a full analysis set that included *n* = 48 placebo and *n* = 49 oxytocin. A total of 15 participants withdrew from the study early after receiving study drug, meaning they were randomized but lost to follow up (*n* = 5 placebo and *n* = 4 oxytocin) or withdrew consent (*n* = 3 placebo and *n* = 3 oxytocin). Overall, 85% of participants completed the intervention phase of the study with no significant differences between oxytocin and placebo (86% vs. 84%, respectively, *p* = 1.00). The first participant was recruited on June 29, 2022, and the last participant completed the study on October 13, 2023.

Participants in the oxytocin and placebo groups were not statistically different on any baseline characteristic (Table 1). Participants were mostly male, White, employed, middle‐aged, married, and high school educated. On average, participants drank heavily, consuming ~54 drinks per week, and met or exceeded four drinks (women) or five drinks (men) per drinking day on ~77% of the days. With respect to treatment drinking goals, ~21% desired abstinence, whereas the majority sought to drink in a limited manner. About 14% smoked at least one cigarette in the week before the screening visit, 17% used other nicotine products, and 33% tested positive for THC. On average, participants had very low levels of alcohol withdrawal (CIWA‐Ar = 1.1). Over one‐quarter (25.8%) met DSM‐5 criteria for major depressive episode in the past year; however, on average, relatively low levels of anxiety and mood disturbance were observed (STAI = 46.2 and POMS Total Mood Disturbance = 7.0, respectively). Sleep quality was in the normal range (PROMIS Sleep Disturbance = 52.6). There was no concomitant care received by either group throughout the trial.

**TABLE 1:** Baseline characteristics of participants (full analysis set).

Measurement	Placebo *N* = 48			Oxytocin *N* = 49			*p* ^a^
	*n*	Mean or %	SD	*n*	Mean or %	SD	
**Demographics**							
Age		51.1	10.1		50.0	10.8	0.612^t^
Gender							0.680^f^
Male	30	62.5%		28	57.1%		
Female	18	37.5%		21	42.9%		
Race/ethnicity							0.108^c^
White	42	87.5%		37	75.5%		
Black	5	10.4%		4	8.2%		
Hispanic	1	2.1%		5	10.2%		
Other	0	0%		3	6.1%		
Education (> high school)	41	85.4%		43	87.8%		1.000^f^
Married	38	79.2%		34	69.4%		0.354^f^
Employed	37	77.1%		41	83.7%		0.453^f^
**Self‐reported alcohol consumption** ^b^							
Drinks per week		54.7	29.6		52.9	40.4	0.280^w^
Drinks per drinking day		8.6	4.2		8.6	5.9	0.391^w^
Percentage of heavy drinking days		79.8	21.5		74.7	23.0	0.262^t^
Percentage of days abstinent		10.2	11.2		11.3	13.9	0.886^w^
World Health Organization (WHO) risk level (grams alcohol per day)							0.518^c^
Moderate risk (men: 41–60, women: 21–40)	1	2.1%		3	6.1%		
High risk (men: 61–100, women: 41–60)	19	39.6%		16	32.7%		
Very high risk (men: > 100, women: > 60)	28	58.3%		30	61.2%		
**Other substance‐related indicators**							
Urge to drink scale (UDS)		17.7	5.8		16.5	5.6	0.296^t^
Drinking goal							
To stop drinking	13	27.1%		7	14.3%		0.139^f^
Reduce drinking but not stop	35	72.9%		42	85.7%		
Alcohol use disorder severity							0.503^f^
Moderate (4–5 symptoms)	15	31.3%		12	24.5%		
Severe (6+ symptoms)	33	68.8%		37	75.5%		
Age onset of regular drinking		18.5	3.5		18.9	4.6	0.675^w^
Inventory of drinking situations (IDS)							
Reward drinker subscale		9.43	3.5		8.6	3.6	0.245^t^
Relief drinker subscale		4.4	3.7		5.3	3.5	0.204^t^
Alcohol use negative consequences (PROMIS)		52.8	4.6		51.7	4.1	0.228^t^
Clinical Institute Withdrawal Assessment of Alcohol Revised (CIWA‐Ar)		1.2	1.7		1.1	2.0	0.606^w^
Smoke cigarettes	8	16.7%		6	12.2%		0.576^f^
Other nicotine use	11	22.9%		5	10.2%		0.108^f^
THC positive urine	17	35.4%		15	30.6%		0.669^f^
**Psychiatric characteristics**							
Other DSM‐5 disorders							
Major depressive episode	15	31.3%		10	20.4%		
Suicidality	3	6.3%		1	2.0%		
Barratt impulsivity (BIS)		67.7	6.0		68.0	7.5	0.632^w^
Spielberger Trait Anxiety Inventory (STAI)		45.9	4.0		46.5	4.4	0.421^w^
Buss Perry Aggression Questionnaire (BPAQ‐SF)		19.9	7.4		18.9	6.9	0.362^w^
Physical aggression		5.1	1.9		5.4	2.3	0.375^w^
Anger		2.9	1.6		2.8	1.5	0.956^w^
Verbal aggression		6.6	3.0		5.8	2.4	0.24^w^
Hostility		5.3	2.8		4.9	2.5	0.432^w^
Attachment‐related anxiety (ECR‐RS)		48.7	22.4		46.8	18.2	0.762^t^
Simplified nutritional appetite questionnaire (SNAQ)		15.1	2.5		15.1	2.8	0.879^w^
Simplified nutritional appetite questionnaire (SNAQ) – ≤ 14	24	50.0%		23	47.9%		1.000^f^
Sleep disturbances (PROMIS)		53.1	9.6		52.1	8.0	0.560^t^
Pain interference (PROMIS)		52.6	9.4		51.1	9.5	0.425^t^
Total mood disturbance (POMS)		6.6	24.8		7.3	23.1	0.801^w^

*Note:* Full analysis set includes participants who took at least one dose of medication. Scale, number of items (range), and interpretive values are as follows: UDS: 5 items (0–35); IDS reward drinker subscale: 5 items (0–15); IDS relief drinker subscale: 5 items (0–15); PROMIS Alcohol Use Negative Consequences—Long Form: 31 items; T‐score; CIWA‐AR: 10 items (0 to 67); ≥ 10 indicative of alcohol withdrawal symptoms that may require treatment.; BIS: 30 items (30–120); STAI: 20 items; T‐score; BPAQ‐SF: 12 items (0–60); physical aggression (4 items), verbal aggression (3 items), anger (2 items), and hostility (3 items); Experiences in Close Relationships—Relationship Structures Questionnaire (ECR‐RS)—attachment‐related anxiety: 18 items (0–126); SNAQ: 4 items (4–20); ≤ 14 indicates significant risk of at least 5% weight loss within 6 months; PROMIS Sleep Disturbances—Short Form 8b: 8 items; T‐score; PROMIS Pain Interference—Short Form 8a: 8 items; T‐score; POMS Total Mood Disturbance: 58 items (−32–200).

^a^ Group mean differences were tested for significance via *t*‐tests of independent samples (t) for normally distributed variables or Wilcoxon rank‐sum tests (w) for skewed variables. Group prevalence rate differences were tested for significance via chi‐square (c) or Fisher's exact tests (f).

^b^ Reflects mean values during the 28‐day period before screening.

^c^ Calculated for participants with a goal of reduced drinking; these participants were asked to estimate, having achieved their drinking goal, the number of drinks they might consume on each day of a typical week at study end.

### 3.2 Medication Compliance

Self‐reported medication compliance during the maintenance phase was similar for both treatment groups (94.7% and 92.7% for oxytocin and placebo groups, respectively; *p* = 0.452). Compliance during the maintenance phase, based on weight of study drug vials before and after use, was also not statistically different when expressed as percentage of prescribed dose (87.7% in each group; *p* = 0.994).

### 3.3 Primary Efficacy Endpoint

**FIGURE 2:**
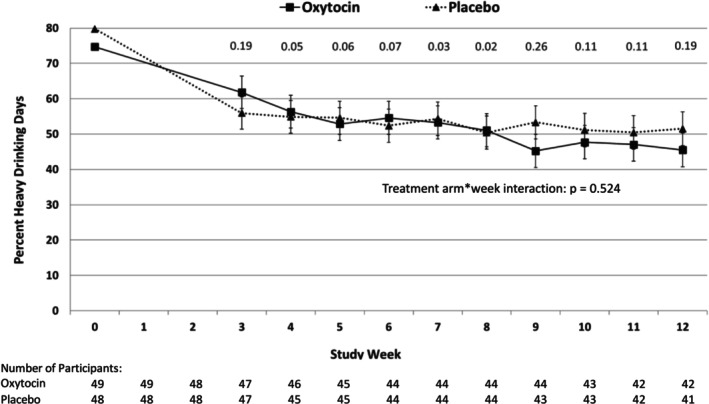
Differences between oxytocin and placebo during the study maintenance phase (Weeks 3–12): Percentage of heavy drinking days. Means are least‐squares means (LSMEANS) from a mixed model. All weekly differences were not significant (*p* > 0.216). Error bars represent standard errors.

Averaged across weeks 3–12 of the maintenance phase, the oxytocin and placebo groups did not statistically differ on the primary efficacy endpoint, weekly PHDD (LSMEANS: 52.9 vs. 51.5, respectively; *d* = 0.04, *p* = 0.798) (Table 2). Similar results were observed with multiple imputation of missing data (48.9 vs. 52.8, respectively; *d* = 0.12, *p* = 0.499). The treatment effect did not differ significantly by week (treatment arm by week interaction, *p* = 0.524). However, PHDD in the oxytocin group did trend downward in Weeks 9–12, compared with the placebo group, though treatment effects were still small and not significant during this period (*d's* = 0.11–0.26; *p*'s = 0.216–0.603) (Figure 2).

**TABLE 2:** Treatment outcomes: differences between placebo and oxytocin during study maintenance phase (Weeks 3–12), full analysis set.

	Placebo *N* = 48			Oxytocin *N* = 49			LSMEAN ∆	SE	*d*	*p*
	LSMEAN	SE	95% CI	LSMEAN	SE	95% CI				
**Drinking outcomes**										
Percent heavy drinking days (primary outcome)										
No Imputation	52.9	3.9	45.2–60.6	51.5	3.9	43.8–59.2	−1.4	5.3	−0.04	0.798
Multiple imputation	52.8	4.1	44.7–60.8	48.9	4.1	40.8–56.9	−3.9	5.8	−0.12	0.499
Drinks per week	34.7	2.3	30.2–39.1	33.1	2.2	28.6–37.5	−1.6	3.1	−0.07	0.681
Drinks per drinking day	6.3	0.3	5.7–6.9	6.2	0.3	5.6–6.8	−0.03	0.4	−0.03	0.855
Percent days abstinent	22.6	2.5	17.6–27.6	23.2	2.5	18.2–28.2	0.61	3.5	0.004	0.980
	**%**	***n***	**Denom**	**%**	***n***	**Denom**	**aOR (95% CI)**			***p*** ^a^
Percent participants abstinent	2.1	1	48	0	0	47				1.000
Percent participants no heavy drinking days	6.3	3	48	2.1	1	47				0.617
Percent participants ≥ WHO 1 level reduction	53.2	25	47	59.6	28	47	1.24 (0.54–2.86)			0.608
Percent participants ≥ WHO 2 level reduction	23.4	11	47	23.4	11	47	0.98 (0.37–2.58)			0.963
**Non‐drinking outcomes**										
THC positive urine^b^	50.0	24	48	49.0	24	49	1.08 (0.35–3.35)			0.888
AUD # of symptoms (MINI)	4.2	0.3	3.6–4.9	3.9	0.3	3.9–4.5	−0.3	0.4	−0.14	0.543
Alcohol use negative consequences (PROMIS)	47.7	0.6	46.5–48.9	48.5	0.6	47.4–49.7	0.9	0.8	0.19	0.310
Sleep disturbances (PROMIS)	51.3	0.7	49.9–52.7	51.0	0.7	49.6–52.4	−0.3	1.0	−0.05	0.786
Pain interference (PROMIS)	52.5	1.0	50.3–54.2	52.2	1.0	50.3–54.2	−0.2	1.4	−0.03	0.858
Urge to drink (UDS)	13.2	0.7	11.7–14.6	13.6	0.7	12.2–15.0	0.4	1.0	0.08	0.674
Total mood disturbance (POMS)	7.6	2.4	2.7–12.5	8.9	2.4	4.1–13.6	1.3	3.3	0.14	0.452
Attachment‐related anxiety (ECR‐RS)	2.4	0.1	2.2–2.6	2.4	0.1	2.2–2.5	−0.03	0.1	−0.07	0.703
	***n***	**Mean**	**SD**	***n***	**Mean**	**SD**				***p***
Cigarettes smoked per week^c^	7	45.9	52.8	4	40.6	32.1				0.854^w^
Other nicotine use (days per week)^d^	8	5.4	2.3	4	4.9	1.9				0.742^t^

*Note:* Models were based on the full analysis set of participants that took one dose of medication. No imputation was used for missing outcome data, unless otherwise specified. For continuous outcomes, LSMEANS were estimated from fully adjusted models on untransformed outcomes (for interpretive purposes); corresponding Cohen's *d* and *p*‐values were based on the same model but with the appropriately transformed outcome.

Abbreviations: ∆, oxytocin – placebo difference; aOR, adjusted odds ratio; CI, confidence interval; *d*, Cohen's *d* (oxytocin‐placebo); Denom, denominator; LSMEAN, least squares mean; SD, standard deviation; SE, standard error; t, *t*‐tests of independent samples; w, Wilcoxon rank‐sum test.

^a^ *p*‐values for dichotomous outcomes are from Fisher's exact tests; except those for WHO level reduction and THC outcomes are from fully adjusted logistic regression.

^b^ Assessed at Week 12.

^c^ Among participants who smoked at baseline (placebo *n* = 8, oxytocin *n* = 6).

^d^ Among participants who used other nicotine products at baseline (placebo *n* = 11, oxytocin *n* = 5).

### 3.4 Secondary Efficacy Endpoints

There were no significant differences between the oxytocin and placebo groups on secondary measures of alcohol consumption, including drinks per week (33.1 vs. 34.7, respectively; *d* = 0.07, *p* = 0.681), drinks per drinking day (6.2 vs., 6.3, respectively; *d* = 0.03, *p* = 0.855), percent days abstinent (23.2 vs. 22.6, respectively; *d* = 0.004, *p* = 0.980), percent participants abstinent (0.0% vs. 2.1%, respectively; *p* = 1.000), and percent participants with no heavy drinking days (2.1% vs. 6.3%, respectively; *p* = 0.617) (Table 2). The low rate of abstinence was expected, as most participants at randomization said their goal was to reduce alcohol consumption, not to stop drinking. There were also no significant differences between the oxytocin and placebo groups in the percentage of participants able to achieve at least a WHO 1‐level reduction (59.6% vs. 53.2%, respectively; *aOR* = 1.24; *p* = 0.608) and at least a WHO 2‐level reduction (23.4% for both groups; *aOR* = 0.98, *p* = 0.963).

Non‐drinking secondary outcomes were not significantly different between the oxytocin and placebo groups during treatment (Table 2). These include THC‐positive urine, AUD number of symptoms (MINI), alcohol use negative consequences (PROMIS), sleep disturbances (PROMIS), pain interference (PROMIS), urge to drink (UDS), attachment‐related anxiety (ECR‐RS), cigarettes smoked per week, days per week of other nicotine use, and total mood disturbance (POMS). In addition, there were also no significant differences between treatment groups on POMS subscales, including tension–anxiety, depression–dejection, anger–hostility, vigor–activity, fatigue–inertia, and confusion–bewilderment (data not shown).

Of the 13 moderators evaluated, none were statistically significant (all treatment arm by moderator interaction *p*'s > 0.128). Although no treatment effects were statistically significant in any subgroup, three moderators produced a subgroup with at least a small effect size favoring oxytocin (*d* ≥ 0.20): number of AUD symptoms (7+ symptoms subgroup: oxytocin = 52.6 vs. placebo = 60.6; *d* = 0.26, *p* = 0.254), IDS reward drinking (high median‐spit subgroup [10+ cutoff]: oxytocin = 48.5 vs. placebo = 57.2; *d* = 0.28, *p* = 0.236), and POMS depression–dejection subscale (high median‐spit subgroup [4+ cutoff]: oxytocin = 54.4 vs. placebo = 61.4; *d* = 0.23, *p* = 0.366). On exit interview, participants in the placebo group were significantly more likely to correctly guess their treatment assignment compared with those in the oxytocin group (54.8% vs. 16.3%, respectively, *p* = 0.0002). Reasons cited were similar between the groups, with most participants listing “no improvement in their drinking” (45.9%) as the reason they believed they received that particular study medication.

### 3.5 Safety

Intranasal oxytocin was generally well tolerated. Most AEs associated with oxytocin were mild (69.9%) or moderate (26.5%), only 0.4% were severe, and none were serious. The prevalence of specific AEs in the oxytocin and placebo groups was not significantly different (Table 3). Hyposmia was the most frequently reported AE, though rates were similar between groups (oxytocin = 55.1% vs. placebo = 52.1%). Only two cases of hyposmia, one for oxytocin and one for placebo, were considered severe. The relatively similar rates of hyposmia (assessed by self‐report and the UPSIT), and the infrequency of severe hyposmia, in both groups suggest that losing the sense of smell may not have been due to oxytocin, but to other factors, such as upper respiratory tract infections, a nocebo effect (given the informed consent form list hyposmia as a potential side effect), inactive ingredients in the nasal spray formulations, or from heavy drinking (Agarwal et al. 2023). Compared to placebo, intranasal oxytocin had numerically higher rates of hypertension, nasopharyngitis, hyperkalemia, cough, bradycardia, nausea, vomiting, hyponatremia, insomnia, and nasal discomfort. No participants reported suicidal ideation or significant alcohol withdrawal symptoms during the study.

**TABLE 3:** Number and percentage of participants with adverse events occurring in at least 5% of participants.

MedDRA preferred term	Placebo (*N* = 48)	Oxytocin (*N* = 49)	*p* ^a^
Hyposmia	25 (52.1%)	27 (55.1%)	0.840
Hypertension	10 (20.8%)	13 (26.5%)	0.634
Nasopharyngitis	3 (6.3%)	9 (18.4%)	0.121
Headache	8 (16.7%)	6 (12.2%)	0.576
Rhinorrhea	7 (14.6%)	3 (6.1%)	0.199
Alanine aminotransferase increased	5 (10.4%)	5 (10.2%)	1.000
Aspartate aminotransferase increased	4 (8.3%)	5 (10.2%)	1.000
Hyperkalemia	3 (6.3%)	5 (10.2%)	0.715
Cough	3 (6.3%)	5 (10.2%)	0.715
Nasal congestion	4 (8.3%)	4 (8.2%)	1.000
Nasal mucosal disorder	4 (8.3%)	2 (4.1%)	0.436
Bradycardia	0 (0.0%)	4 (8.2%)	0.117
Nausea	0 (0.0%)	4 (8.2%)	0.117
Vomiting	0 (0.0%)	3 (6.1%)	0.242
Hyponatremia	0 (0.0%)	3 (6.1%)	0.242
Insomnia	0 (0.0%)	3 (6.1%)	0.242
Nasal discomfort	0 (0.0%)	3 (6.1%)	0.242
Diarrhea	3 (6.3%)	0 (0.0%)	0.117
Upper respiratory tract infection	3 (6.3%)	0 (0.0%)	0.117
Decreased appetite	3 (6.3%)	0 (0.0%)	0.117
Hypernatremia	3 (6.3%)	0 (0.0%)	0.117
Erythema	3 (6.3%)	0 (0.0%)	0.117

*Note:* Multiple occurrences of a specific adverse event for a participant were counted once in the frequency for the adverse event. Adverse events with at least 5% of participants occurring in either arm were included in the table, sorted by total prevalence.

^a^ Fisher's exact test.

Participants reported little change in appetite as mean SNAQ scores were approximately 15 for participants at baseline in both groups and remained unchanged throughout the study. The groups also had similar percentages of participants with SNAQ scores ≤ 14 by week 13 (oxytoci*n* = 8.2% vs. placebo = 6.3%; *p* = 0.716). Total BPAQ‐SF scores did not statistically differ between groups across the treatment period (oxytocin = 17.8 vs. placebo = 18.2; *d* = 0.12, *p* = 0.500).

## 4 Discussion

The purpose of this 12‐week randomized, placebo‐controlled clinical trial was to evaluate the efficacy and safety of intranasal oxytocin in individuals with at least moderate AUD. There were no statistically significant differences in the primary or secondary drinking measures between the oxytocin and placebo groups over the 10‐week maintenance period of the study (Weeks 3–12). Interestingly, at the end of the study, between Weeks 9 and 12, drinking steadily declined in the oxytocin group, whereas the placebo group's drinking remained the same. Although the differences were not significant during this period and treatment effects were modest, this trend toward reduced drinking, if continued, suggests that a longer treatment period (greater than 12 weeks) may be needed to realize oxytocin's optimal effects, if indeed there is efficacy. In addition, there were no significant differences between treatment groups on the non‐drinking outcomes.

Candidate compounds in AUD pharmacotherapy trials often exhibit only a small effect size or no significant effects on drinking outcomes. Over the past three decades, more than 30 different compounds, with varied molecular targets, have been tested in randomized controlled trials for AUD and shown either no significant effect or a small effect size (< 0.5) (Jonas et al. 2014; Kranzler and Hartwell 2023; Litten et al. 2018). These results are likely due to AUD's heterogeneity, which is marked by a wide range of genetic and environmental influences (Litten et al. 2015), and the placebo effect, a common phenomenon in all AUD trials, including this current study (Litten et al. 2013).

Precision medicine, which seeks to identify and target specific phenotypes that are most likely to respond favorably to a given medication, should help address AUD's heterogeneity. Indeed, the current study explored 13 potential moderators of treatment effect. While statistical power was modest for this purpose, and hence, no statistical differences were found, small effect sizes favoring oxytocin were observed among individuals with severe AUD, motivated by reward drinking, and greater depression. Identifying potential biomarkers of oxytocin's effects are in early stages. For example, human studies have shown that the single nucleotide polymorphisms (SNPs) rs4564970 and rs1488467, located within the oxytocin receptor gene (OXTR), are associated with altered oxytocin signaling and physiological responses (Jern et al. 2012; Johansson et al. 2012). More recently, emerging evidence has identified genetic variation within the oxytocin gene itself, specifically the OXT rs6133010 variant, as a potential moderator of psychiatric symptoms during alcohol withdrawal. Specifically, G allele carriers exhibit heightened hostility and anxiety in individuals with alcohol use disorder (AUD), whereas AA homozygotes appear to have a protective effect. These findings suggest that oxytocin's therapeutic effects may be influenced by genetic factors, which could explain why some individuals respond to oxytocin treatment while others do not. To advance precision medicine, future studies need to explore other biomarkers that could be linked to oxytocin by examining multi‐omics, brain and cell imaging, and electrophysiological variations. Advanced analytical approaches, coupled with artificial intelligence and machine learning, will play an important role in defining different phenotypes and identifying individuals who are most likely to respond to a given medication (Litten et al. 2020).

Efforts were made to minimize the placebo effect in this study. Participants were required to have at least one heavy drinking day within 7 days before randomization, excluding those who had already stopped drinking. Additionally, the *Take Control* behavioral platform was used, providing a standardized but modest treatment effect (Devine et al. 2016). Despite these measures, both treatment groups showed reduced drinking during treatment—a common challenge in AUD pharmacotherapy trials that complicates the detection of significant medication effects.

This study's strengths include a multi‐site trial of four clinical sites, which were rigorously monitored throughout the study by a clinical research organization. Other strengths include high medication adherence and treatment retention, low drop‐out rate and missing data, and use of a standardized adjunctive behavioral treatment. Moreover, study procedures to maximize blinding appear effective as participants were not better than chance at guessing their treatment assignment; however, it is possible that intranasal administration of oxytocin may have produced local sensory effects to permit treatment inference and possibly affect subjective outcomes and adverse event reporting. Limitations include the lack of statistical power to detect small differences between the oxytocin and placebo groups as well as possible moderators of the oxytocin effect on drinking, such as gender or baseline alcohol consumption. In addition, individuals with significant alcohol withdrawal and psychiatric comorbidity, which are common in AUD outpatient settings, were excluded from the study, limiting generalizability. Plasma oxytocin levels were not measured. Although alcohol consumption was assessed using a well‐validated self‐report method (TLFB), recall or social desirability bias is possible and objective biomarkers, such as PEth or CDT, were not captured and available for corroboration. Finally, completion of Take Control modules was not recorded during each visit.

In summary, oxytocin was well tolerated and adverse events were generally similar in both the treatment and placebo groups. Both the intranasal oxytocin and placebo groups significantly reduced their drinking at the end of the treatment period, compared with baseline levels. Although no significant differences were found between the two groups in the primary and secondary drinking endpoints, drinking steadily declined in the later weeks of the study in participants treated with oxytocin, whereas those treated with placebo leveled off. This suggests that a longer course of treatment might be needed to realize oxytocin's maximal effects, if oxytocin has efficacy. It is also possible that oxytocin had a small effect that was masked by the placebo effect. Finally, it is possible that the dose of 70 IU/day was insufficient to reduce alcohol consumption and that higher doses are necessary to achieve greater treatment effects. Additional studies should focus on determining the optimal dosing and treatment duration as well as identifying specific patient subgroups that might derive the greatest benefit from oxytocin treatment.

## Funding

This study was funded by the National Institute on Alcohol Abuse and Alcoholism (NIAAA) under Contract HHSN275201400001I. The study was designed collaboratively by NIAAA, the clinical site principal investigators, and FastTrack. FastTrack monitored study progress and oversaw data collection. NIAAA was responsible for data analysis and interpretation in collaboration with the clinical site principal investigator and FastTrack. This study was sponsored by NIAAA and National Institutes of Health (NIH) Division of Medications Development. The investigational products were provided by The University of Iowa.

## Conflicts of Interest

The authors declare no conflicts of interest.

## Supporting information


**Appendix S1:** Inclusion/exclusion criteria.
**Appendix S2:** Assessment schedule.
**Appendix S3:** Statistical analysis details.

## Data Availability

Individual de‐identified participant data—including the data dictionary, statistical code, and other study materials—will be made available by NIAAA through controlled access. Qualified investigators may request access, which will be granted upon approval by the NIAAA Data Access Committee and execution of the controlled access data use agreement.
