# Supplementary material for: Intranasal Oxytocin for Alcohol Use Disorder: A Randomized, Double‐Blind, Placebo‐Controlled Multisite Trial Assessing Efficacy and Safety
Source: Alcohol Clin Exp Res (Hoboken). 2026 Jul 15;50(7):e70326. doi: 10.1111/acer.70326 (PMC13373018; doi:10.1111/acer.70326)
Supplement: Supplementary file 1 — Appendix S1: Inclusion/exclusion criteria. Appendix S2: Assessment schedule. Appendix S3: Statistical analysis details. [file ACER-50-0-s001.docx]

**Supplementary Appendix 1: Inclusion/Exclusion Criteria**

Inclusion Criteria

Subjects must have met each one of the following inclusion criteria in order to be eligible for participation in the study:

1. Be at least 21 years of age.
2. Have a current (past 12 months) DSM-5 diagnosis of AUD (4 or more symptoms) assessed using the MINI neuropsychiatric interview version 7.0.2 (at least moderate severity, ICD-10-CM Code F10.20 alcohol dependence, uncomplicated).
3. If male, report drinking an average of at least 28 drinks per week or if female report drinking an average of at least 21 drinks per week and at least one heavy drinking day per week for the 28-day period prior to consent and at least one heavy drinking day in the 7‑ day period prior to randomization.
4. Have a BAC by breathalyzer equal to 0.000 when s/he signed the informed consent document (either just prior to or immediately after signing consent).
5. Be seeking treatment for problems with alcohol and express a goal of abstinence or a reduction in drinking.
6. Be able to verbalize an understanding of the consent form, able to provide written informed consent, verbalize willingness to complete study procedures, able to understand written and oral instructions in English and able to complete the questionnaires required by the protocol.
7. Agree (if the subject is female and of childbearing potential) to use at least one of the following methods of birth control, unless she is surgically sterile, partner is surgically sterile or she is postmenopausal:
   - oral contraceptives,
   - contraceptive sponge,
   - patch,
   - double barrier (diaphragm/spermicidal or condom/spermicidal),
   - intrauterine contraceptive system,
   - etonogestrel implant,
   - medroxyprogesterone acetate contraceptive injection,
   - complete abstinence from sexual intercourse, and/or
   - hormonal vaginal contraceptive ring.
8. Be able to take intranasal investigational products and be willing to adhere to the investigational product regimen.
9. Complete all assessments required at screening and baseline.
10. Have a place to live in the 2 weeks prior to randomization and not be at risk that s/he will lose his/her housing by Study Week 15.
11. Not anticipate any significant problems with transportation arrangements or available time to travel to the study site by Study Week 15.
12. Not have any plans to move within Study Week 15 to a location which would make continued participation in the study impractical.
13. Not have any unresolved legal problems that could jeopardize continuation or completion of the study.
14. Provide contact information of someone, such as a family member, spouse, or significant other, who may be able to contact the subject in case of a missed clinic appointment.
15. Be someone who in the opinion of the investigator would be expected to complete the study protocol.
16. Agree to the schedule of visits, verbally acknowledge that s/he will be able to attend each scheduled visit, participate in phone visits and that s/he does not have any already scheduled events or a job that may substantially interfere with study participation.
17. If taking a medication for depression or anxiety, must have been taking a stable dose in the 2-months prior to randomization and plan to continue during the study. This includes drugs such as the following: SSRIs, Dual uptake inhibitors, SNRIs, Tricyclic antidepressants, MAOIs, and bupropion.
18. Not currently taking oxytocin and agree not to take non-study oxytocin for the duration of the study.
19. Agree to not use nicotine nasal spray for the duration of the study. Note: other forms of nicotine replacement therapy (gum, patch, etc., are permitted).

Exclusion Criteria

A subject was not eligible to participate in this study if any one of the following exclusion criteria was met:

1. Have current substance use disorder for any psychoactive substance (including sedatives and hypnotics) other than alcohol, nicotine or mild marijuana use disorder as defined by DSM-5 criteria.

2. Have a urine toxicology screen positive during screening or baseline for any of the following substances:

- - benzodiazepines,
  - cocaine,
  - opioids,
  - amphetamines,
  - buprenorphine,
  - methadone,
  - methamphetamines
  - oxycodone,
  - MDMA, and/or
  - barbiturates.

Note: Testing for tetrahydrocannabinol (THC) will be included in the urine drug test; however, subjects who test positive for THC are still eligible to participate in the study unless they endorse moderate or severe substance use disorder for marijuana as indicated by DSM-5 criteria. The results for THC will be recorded for information only. If positive for opioids but recent opiate use for acute pain is reported by the subject, then the subject can be re-screened.

3. Have been hospitalized for alcohol intoxication delirium, alcohol withdrawal delirium, alcohol-induced persisting dementia or amnestic disorder, or have had an alcohol withdrawal seizure, alcohol-induced psychotic disorder with a primary diagnosis of alcohol use disorder or a history of any seizure disorder.

4. Have participated (received treatment) in any behavioral and/or pharmacological intervention research study for the treatment of alcohol problems in the past 7 years.

5. Be mandated by the court to obtain treatment for problems with alcohol.

6. Be anyone who in the opinion of the investigator could not be safely withdrawn from alcohol without medical detoxification.

7. Be currently undergoing psychotherapy by a licensed therapist or psychiatrist for alcohol problems

NOTE: Current psychotherapy should be considered on a case-by-case basis. Psychotherapy for a disorder that may be related to the subject’s use of alcohol should be exclusionary. However, shorter term focused behavioral therapy for defined, non-alcohol related problems may be acceptable.

8. Have undergone medical detoxification (e.g., reports using a benzodiazepine) during the screening phase (prior to randomization).

9. Have known allergy to oxytocin.

10. Have significant medical conditions (e.g., chronic rhinitis during the past year) that may preclude successful insufflations of the intranasal investigational products or other medical conditions (e.g., nasal lesions) that could be affected by repeated intranasal administrations.

11. Have been treated with a pharmacotherapy for alcohol problems within 6 months prior to randomization.

12. Have taken any anti-convulsants, hypnotics, barbiturates, antipsychotics, psychomotor stimulants (such as methylphenidate), or benzodiazepines within 5-half lives days prior to the date of randomization.

13. Have any of the following, based on DSM-5 criteria as assessed using the MINI:

- - Current or lifetime diagnosis of psychotic disorders
  - Current bipolar disorder

Note: Subjects diagnosed with psychiatric disorders not specifically excluded above may be excluded at the discretion of the PI if the comorbid psychiatric condition compromises the study integrity by virtue of its type, duration, or intensity.

14. Have any of the following:

- - attempted suicide past year,
  - current (past year) suicide behavior disorder in accordance with DSM-5 criteria as assessed using the MINI (see note below about assessment of subjects diagnosed at low risk), or
  - current (since screening MINI) suicidality risk as indicated during the conduct of the C-SSRS with concurrence after a study physician’s evaluation if the response to C‑SSRS questions 1 or 2 is “yes”).

Note: The MINI suicidality module rates scores of 1 to 8 as a diagnosis of low risk of suicidality. As the MINI questions that could result in a low risk score are considered inadequate to fully determine the potential suicidal risk of an individual (e.g., “Feel hopeless” and “Think that you would be better off dead or wish you were dead?” responses of “yes” dictates a score of 1 for each question), any subject who scores in the low risk category should be evaluated further by a study physician or psychologist who should document whether the subject is appropriate for study inclusion based on his/her clinical judgment of the potential suicide risk of the subject. Likewise, if the subject responds “yes” to either of the first two questions on the screening C-SSRS performed on the day of randomization as a final eligibility check, the subject should also be evaluated by a study physician for current suicidality risk, who should document the subject’s suitability for study inclusion.

15. Have moderate or serious dementia as assessed by clinical exam.

16. Be pregnant or breast-feeding or have plans to become pregnant at any time during the study.

17. Have clinically significant abnormal liver enzyme levels defined as AST or ALT 5-fold above the upper limit of normal (ULN), or bilirubin greater than 2 times the ULN.

Note: If the subject has values of liver enzyme that are 3.0-to-4.9 fold above the ULN and bilirubin that is 1.5-to-1.9 fold above the ULN of normal, these assessments should be repeated at least a week apart and if still in this range or higher, the subject should be excluded from the study and referred to their physician for further follow-up.

1. Have sodium < 132 mmol/L or > 150 mmol/L or potassium < 3.2 mmol/L or > 5.5 mmol/L or abnormal calculated creatinine clearance (<60 mL/min), as calculated by Cockcroft and Gault formula.
2. Have a serious or unstable medical illness or any potentially life-threatening or progressive medical condition other than addiction that may compromise subject safety or study conduct.
3. Have clinically significant gastrointestinal, neurological, renal, or cardiovascular disease such cardiac arrhythmia, uncontrolled hypertension, congestive heart failure, or any other ECG abnormality considered clinically significant by the PI.
4. Have data suggesting cirrhosis of the liver.
5. Have taken oxytocin during the 6-month period prior to randomization for treatment of any disorder or if ever treated with oxytocin for AUD.
6. Have an UPSIT score ≤ 30 in men and ≤31 in women at screening.

**Supplementary Appendix 2: Assessment Schedule**

|  | **Screen** |  | | **Maintenance** | | | | | | | | | | **EOS**^a^ | **Safety Follow-up** |
| --- | --- | --- | --- | --- | --- | --- | --- | --- | --- | --- | --- | --- | --- | --- | --- |
| **Study Week** | **-2 to -1** | **1** | **2** | **3** | **4** | **5** | **6** | **7** | **8** | **9** | **10** | **11** | **12** | **13** | **14/15** |
| **Clinic Visit #** | **1** | **2** | **3** | **4** | **5** |  | **6** |  | **7** |  | **8** |  |  | **9** |  |
| Informed Consent | **X** |  |  |  |  |  |  |  |  |  |  |  |  |  |  |
| Alcohol Breathalyzer | **X** | **X** | **X** | **X** | **X** |  | **X** |  | **X** |  | **X** |  |  | **X** |  |
| Urine Drug Screen^b^ | **X** | **X** | **X** | **X** | **X** |  | **X** |  | **X** |  | **X** |  |  | **X** |  |
| Locator Form | **X** |  |  |  |  |  |  |  |  |  |  |  |  |  |  |
| Demographics | **X** |  |  |  |  |  |  |  |  |  |  |  |  |  |  |
| Medical/Surgical History | **X** | **X**^c^ |  |  |  |  |  |  |  |  |  |  |  |  |  |
| Physical Exam | **X**^d^ | **X** | **X** | **X** | **X** |  | **X** |  | **X** |  | **X** |  |  | **X** |  |
| MINI V 7.0.2 (AUD module at EOS) | **X** |  |  |  |  |  |  |  |  |  |  |  |  | **X** |  |
| C-SSRS |  | **X** | **X** |  |  |  | **X** |  | **X** |  | **X** |  |  | **X** |  |
| Clinical Chemistry^e^ | **X** |  |  |  |  |  | **X** |  | **X** |  | **X** |  |  | **X** |  |
| Vital Signs | **X** | **X** | **X** | **X** | **X** |  | **X** |  | **X** |  | **X** |  |  | **X** |  |
| ECG (12-lead) | **X** |  |  |  |  |  |  |  |  |  |  |  |  | **X** |  |
| Prior and Concomitant Meds | **X** | **X** | **X** | **X** | **X** | **X** | **X** | **X** | **X** | **X** | **X** | **X** | **X** | **X** | **X** |
| CIWA-AR | **X** | **X** | **X** | **X** | **X** |  | **X** |  | **X** |  | **X** |  |  | **X** |  |
| Eligibility Checklist | **X** | **X**^c^ |  |  |  |  |  |  |  |  |  |  |  |  |  |
| Drug Compliance – Diary review |  | **X** | **X** | **X** | **X** |  | **X** |  | **X** |  | **X** |  |  | **X** |  |
| Drug Accountability (vial weight) |  | **X** | **X** | **X** | **X** |  | **X** |  | **X** |  | **X** |  |  | **X** |  |
| Pregnancy Test/Female Birth Control Methods | **X** | **X** | **X**^f^ |  |  |  | **X** |  | **X** |  | **X** |  |  | **X** |  |
| Weight | **X** |  |  |  |  |  | **X** |  | **X** |  | **X** |  |  | **X** |  |
| Drinking Goal | **X** |  |  |  |  |  |  |  |  |  |  |  |  |  |  |
| AEs/SAEs |  |  | **X** | **X** | **X** | **X** | **X** | **X** | **X** | **X** | **X** | **X** | **X** | **X** | **X** |
| Other Services Used for Alcohol Use Problems^g^ |  | **X** |  |  |  |  |  |  |  |  |  |  |  | **X** |  |
| **RANDOMIZATION** |  | **X** |  |  |  |  |  |  |  |  |  |  |  |  |  |
| Brief Telephone Interview^h^ |  |  |  |  |  | **X** |  | **X** |  | **X** |  | **X** | **X** |  |  |
| Take Control |  | **X** | **X** | **X** | **X** |  | **X** |  | **X** |  | **X** |  |  |  |  |
| Exit Interview |  |  |  |  |  |  |  |  |  |  |  |  |  | **X** |  |
| Treatment Referral |  |  |  |  |  |  |  |  |  |  |  |  |  | **X** |  |
| Follow-Up Telephone Interview |  |  |  |  |  |  |  |  |  |  |  |  |  |  | **X** |
| Final Subject Disposition |  |  |  |  |  |  |  |  |  |  |  |  |  |  | **X** |
| **Subject Reported Outcomes** |  |  |  |  |  |  |  |  |  |  |  |  |  |  |  |
| Hyperkatefia Scale |  | **X** |  |  |  |  |  |  |  |  |  |  |  | **X** |  |
| Barrett Impulsivity Scale |  | **X** |  |  |  |  |  |  |  |  |  |  |  |  |  |
| Spielberger Trait Anxiety Inventory |  | **X** |  |  |  |  |  |  |  |  |  |  |  |  |  |
| BPAQ - SF^i^ |  | **X** |  |  |  |  | **X** |  | **X** |  | **X** |  |  | **X** |  |
| Simplified Nutritional Appetite Questionnaire |  | **X** |  |  |  |  | **X** |  | **X** |  | **X** |  |  | **X** |  |
| Cigarette and other nicotine use |  | **X** |  |  |  |  | **X** |  | **X** |  | **X** |  |  | **X** |  |
| ECR-RS^j^ |  | **X** |  |  |  |  | **X** |  | **X** |  | **X** |  |  | **X** |  |
| IDS-30^k^ |  | **X** |  |  |  |  |  |  |  |  |  |  |  |  |  |
| POMS |  | **X** |  |  |  |  | **X** |  | **X** |  | **X** |  |  | **X** |  |
| PROMIS – alcohol negative consequences |  | **X** |  |  |  |  | **X** |  | **X** |  | **X** |  |  | **X** |  |
| PROMIS – sleep disturbances |  | **X** |  |  |  |  | **X** |  | **X** |  | **X** |  |  | **X** |  |
| PROMIS – pain interference |  | **X** |  |  |  |  | **X** |  | **X** |  | **X** |  |  | **X** |  |
| UPSIT^l^ | **X** |  |  | **X** |  |  | **X** |  | **X** |  | **X** |  |  | **X** |  |
| Urge to drink questionnaire |  | **X** |  |  |  |  | **X** |  | **X** |  | **X** |  |  | **X** |  |
| Timeline followback (TLFB) | **X** | **X** | **X** | **X** | **X** |  | **X** |  | **X** |  | **X** |  |  | **X** |  |
| Brief Drinking Questionnaire^m^ |  |  |  |  |  |  |  |  |  |  |  |  |  | **X** |  |

^a^ EOS=end of study. These assessments are to be done at Week 13 or if the subject discontinues early and agrees to a final clinic visit.

^b^ Test for opioids, cocaine, amphetamines, methamphetamine, THC, buprenorphine, methadone, benzodiazepines, oxycodone, 3,4-methylenedioxy-methamphetamine (MDMA), and barbiturates.

^c^ Updated prior to randomization.

^d^ Complete physical exam at screening including examination of the nares, plus examination of the nares at all other visits as indicated.

^e^ AST, ALT, total bilirubin, creatinine, sodium, and potassium.

^f^ Only birth control methods are collected at this visit.

^g^ At baseline asks about lifetime treatment use and at EOS asks “since beginning this study

^h^ AEs, concomitant medications, and drug compliance reminder.

^i^ Buss Perry Aggression Questionnaire – Short Form

^j^ Experiences in Close Relationships—Relationship Structures Questionnaire (ECR-RS) (attachment related anxiety)

^k^ Inventory of Drinking Situations

^l^ University of Pennsylvania Smell Identification Test

^m^ Only asked to subjects who request to withdraw from the study and are not willing to provide TLFB drinking data. This questionnaire will be asked at whatever constitutes the EOS visit.

**Supplementary Appendix 3: Statistical Analysis Details**

Continuous drinking endpoints were summarized per week and consisted of percent heavy drinking days (PHDD; the primary outcome), number of drinks per week, drinks per drinking day, and percent days abstinent. To allow for modeling, each continuous drinking endpoint was evaluated for normality. The following endpoints were transformed: drinks per week (square root), drinks per drinking day (log), percent days abstinent (square root). Each of these endpoints was analyzed using a mixed effects (ME) model for repeated measures employing the Toeplitz covariance structure which had the best fit compared to compound symmetry and unstructured. Each ME model included fixed effects for the treatment group, study week, treatment group by study week interaction, clinical site, baseline equivalent of the endpoint, and consecutive days abstinent in the screening period prior to randomization. Subject-level variability was treated as a random effect in the ME model.

For the PHDD outcome, a sensitivity analysis using multiple imputation was used to handle missing data. The same variables used in the ME model were included in the multiple imputation model, plus time-related variables (i.e., the endpoint prior to dropout). Each of the “m” imputed datasets were analyzed using ME models, then SAS PROC MIANALYZE to combine the parameter estimates.

Other repeated continuous outcomes – POMS (except Anger-Hostility subscale), PROMIS measures (alcohol use negative consequences, sleep disturbances, and pain interference), ECR-RS, urge to drink, and SNAQ total score – were analyzed the same ME model as that continuous drinking endpoints, with minor differences in covariates. For instance, the covariate, consecutive days abstinent in the screening period prior to randomization, was not included in these models. The ME model for the PROMIS measures included the participant’s age at study entry as an additional fixed effect. POMS subscales, except Anger and Vigor, used a log transformation. Inverse transformation was used in ECR-RS. Cigarettes smoked per week and days per week of other nicotine use were calculated and analyzed only among individuals who smoked or had other nicotine use at baseline, respectively, and were not modeled due to the small numbers of participants.

The continuous outcome, number of AUD symptoms, was analyzed using am analysis of covariance (ANCOVA) that included treatment arm, site and baseline AUD number of symptoms.

For dichotomous outcomes, unadjusted percentages were presented. Additional analyses of the dichotomous outcomes are as follows. The POMS Anger-Hostility subscale was dichotomized as 0 versus 1+, due to high skew. BPAQ-SF subscales were skewed and, thus, dichotomized for multivariate analysis: physical aggression (4 vs 5+), verbal aggression (<6 vs 6+), anger (2 vs 3+), and hostility (3 vs 4+). These outcomes were analyzed using a generalized linear model with logit link using the same covariates as for the other POMS models. LSMEANS, Cohen’s d and p-values were reported from these fully adjusted models. The two dichotomous outcomes – at least a 1- and 2-level reduction in WHO drinking risk level from baseline – were analyzed using a logistic regression model that included treatment arm and clinical site. Adjusted odds ratios, their 95% confidence intervals, and p-values were presented. The dichotomous outcome, THC positive urine, was coded as “1” if any of the urine assessments during the maintenance period showed positive THC use, else “0”. This outcome was analyzed using a logistic regression model that included, treatment arm, clinical site, and THC positive urine at baseline. The outcomes percent participants with no heavy drinking days percent participants abstinent and SNAQ score (dichotomized as <14 vs 14+) were not modeled due to the small number of participants in these groups.

Changes in UPSIT score were reported as adverse events. Adverse event data were presented by frequency.

Exploratory moderation analyses were conducted for PHDD outcome to evaluate whether a differential treatment effect existed for 13 patient characteristics of theoretical and scientific interest. These characteristics were measured at baseline and included: drinks per week, number of AUD symptoms (MINI), IDS (relief and reward drinking subscales), gender, attachment-related anxiety (ECR-RS), POMS subscales of depression-dejection and tension-anxiety, STAI, BIS (self-control and impulsive behavior subscales), history of alcohol withdrawal (MINI), and drinking goal. No other moderators were evaluated.

To facilitate interpretation, continuous moderator variables were dichotomized via median splits, except where indicated^*^, to maximize sufficient sample size per moderator-by-treatment group for stable estimates. On this basis, the following cutoffs were used:

- Drinks per week: <49 vs >49
- Number of AUD symptoms^*^: moderate AUD (4-6 symptoms) vs severe AUD (7+ symptoms)
- IDS relief drinking subscale: < 5 vs >5
- IDS reward drinking subscale: < 10 vs >10
- Gender: female vs male
- Attachment-related anxiety (ECR-RS): < 2.55 vs >2.55
- POMS depression-dejection subscale: < 3 vs >4
- POMS tension-anxiety subscale: < 4 vs >4
- STAI: < 46 vs >47
- BIS self-control subscale: < 11 vs >11
- BIS impulsive behavior subscale: < 46 vs >47
- History of alcohol withdrawal (MINI): yes vs no
- Drinking goal: abstinence vs reduced drinking

Continuous moderators were checked for non-linearities in the moderated treatment effects via a predicted probability curve obtained from a logistic regression that used only treatment group, variable, and treatment group by variable interaction. The predicted probability for the primary endpoint was plotted for each treatment group across values of the moderator. Moderation analyses used the same ME model as the primary outcome (i.e., PHDD without imputation during the 10-week maintenance treatment period [Study Weeks 3-12]) with the addition of a treatment*moderator interaction term.

For descriptive statistics, group mean differences were tested for significance by t-tests for independent samples for normally distributed variables or Wilcoxon rank-sum tests for skewed variables. Group prevalence rate differences were tested for significance via chi-square or Fisher’s exact tests.

Cohen’s d = (µ_Treatment_ – µ_Placebo_) /σ, where µ_Treatment_ – µ_Placebo_ is the difference between the means for the treatment and placebo groups, and σ is the pooled standard deviation. The following are offered as cut-offs for interpreting the effect size: small=.20, medium=.50, and large=.80 (Cohen, 1992).

**References for Supplementary Appendices**

Cohen JA (1992) A power primer. Psychol Bull 112:155-159.
